# Acral cutaneous malignant melanoma treated with linear accelerator-based boron neutron capture therapy system: a case report of first patient

**DOI:** 10.3389/fonc.2023.1272507

**Published:** 2023-10-13

**Authors:** Hiroshi Igaki, Satoshi Nakamura, Naoya Yamazaki, Tomoya Kaneda, Mihiro Takemori, Tairo Kashihara, Naoya Murakami, Kenjiro Namikawa, Tetsu Nakaichi, Hiroyuki Okamoto, Kotaro Iijima, Takahito Chiba, Hiroki Nakayama, Ayaka Nagao, Madoka Sakuramachi, Kana Takahashi, Koji Inaba, Kae Okuma, Yuko Nakayama, Kazuaki Shimada, Hitoshi Nakagama, Jun Itami

**Affiliations:** ^1^ Department of Radiation Oncology, National Cancer Center Hospital, Tokyo, Japan; ^2^ Division of Research and Development for Boron Neutron Capture Therapy, National Cancer Center Exploratory Oncology Research & Clinical Trial Center, Tokyo, Japan; ^3^ Division of Radiation Safety and Quality Assurance, National Cancer Center Hospital, Tokyo, Japan; ^4^ Medical Physics Laboratory, Division of Health Science, Graduate School of Medicine, Osaka University, Suita, Osaka, Japan; ^5^ Department of Dermatologic Oncology, National Cancer Center Hospital, Tokyo, Japan; ^6^ Department of Radiation Oncology, Jutendo University School of Medicine, Tokyo, Japan; ^7^ Department of Radiological Science, Graduate School of Human Health Sciences, Tokyo Metropolitan University, Tokyo, Japan; ^8^ National Cancer Center Hospital, Tokyo, Japan; ^9^ Shin-Matsudo Accuracy Radiation Therapy Center, Shin-Matsudo Central General Hospital, Chiba, Japan

**Keywords:** boron neutron capture therapy (BNCT), malignant melanoma, linear accelerator-based BNCT system, solid-state Li target, acral malignant melanoma, first treatment

## Abstract

This study reports the first patient treatment for cutaneous malignant melanoma using a linear accelerator-based boron neutron capture therapy (BNCT) system. A single-center open-label phase I clinical trial had been conducted using the system since November 2019. A patient with a localized node-negative acral malignant melanoma and the largest diameter of the tumor ≤ 15 cm who refused primary surgery and chemotherapy was enrolled. After administering boronophenylalanine (BPA), a single treatment of BNCT with the maximum dose of 18 Gy-Eq delivered to the skin was performed. The safety and efficacy of the accelerator-based BNCT system for treating localized cutaneous malignant melanoma were evaluated. The first patient with cutaneous malignant melanoma *in situ on* the second finger of the left hand did not develop dose-limiting toxicity in the clinical trial. After BNCT, the treatment efficacy was gradually observed, and the patient achieved PR within 6 months and CR within 12 months. Moreover, during the follow-up period of 12 months after BNCT, the patient did not exhibit a recurrence without any treatment-related grade 2 or higher adverse events. Although grade 1 adverse events of dermatitis, dry skin, skin hyperpigmentation, edema, nausea, and aching pain were noted in the patient, those adverse events were relieved without any treatment. This case report shows that the accelerator-based BNCT may become a promising treatment modality for cutaneous malignant melanoma. We expect further clinical trials to reveal the efficacy and safety of the accelerator-based BNCT for cutaneous malignant melanoma.

## Introduction

Boron neutron capture therapy (BNCT) using an accelerator-based neutron source is one of the recently developed radiotherapies. The principle of therapeutic efficacy is primarily based on ^10^B(n, α)^7^Li reactions. Those particles exhibit high linear energy transfer, and the relative biological effectiveness of BNCT is greater than that of conventional radiotherapies (1). As the ranges of those particles within the human body are comparable to the size of the target cells, when the concentration of ^10^B particles is several times higher in the tumor than in the surrounding normal tissues, selective tumor cell killing can be anticipated. Thus, the higher biological effectiveness via boron neutron capture reaction has been experimentally explored (1).

Clinical trials for BNCT have been performed in research reactors, and favorable clinical outcomes have been reported (2, 3). However, the necessity of using a nuclear reactor as a neutron source posed regulatory and safety concerns that prevented the widespread application of BNCT in oncology practice. Recent researches have resulted in implementing accelerator-based neutron sources for BNCT that can be utilized in hospitals (4, 5). Therefore, the linear accelerator-based BNCT system with a lithium target was installed at the National Cancer Center Hospital (NCCH), Tokyo, Japan, and has been in clinical use following the completion of various preclinical studies (5). A phase I clinical trial has been conducted using this system for treating localized cutaneous malignant melanoma and angiosarcoma since November 2019 (6). As neutrons have a limited penetration depth due to the use of epithermal neutron beam, superficial tumors such as cutaneous malignant melanoma are good candidates for BNCT (7).

Standard treatment for cutaneous malignant melanoma without metastasis is primary surgery with extensive resection, with or without postoperative adjuvant therapy depending on its stage (8). Because malignant melanoma is frequently observed in the extremities and is affected by elderly patients (9), radical resection significantly diminishes the quality of life regarding limb function preservation. Therefore, developing treatment options is expected as an alternative to surgery. Boronophenylalanine (BPA, borofalan(^10^B)), a commonly utilized ^10^B-containing drug in BNCT, is taken up by tumor cells via the L-type amino acid transporter 1 (LAT-1) (10), and cutaneous malignant melanoma expresses LAT-1 at high levels (11). Therefore, cutaneous malignant melanoma was chosen as one of the target diseases for the clinical trial.

This report describes the first patient treatment for cutaneous malignant melanoma using an accelerator-based BNCT system.

## Case description

### Clinical trial overview

The trial utilizes an investigational device (CICS-1, neutron irradiation device manufactured by Cancer Intelligence Care Systems, Inc.) and an investigational drug (SPM-011, borofalan(^10^B) provided by Stella Pharma Corporation) (6). The trial is a single-center, open-label study. The primary endpoint is to assess the incidence of dose-limiting toxicity at a predetermined radiation dose. The severity of adverse events was evaluated based on CTCAE v 5.0. The dose-limiting toxicity was defined as any treatment-related grade 4 or higher and serious adverse event requiring hospitalization occurring within 90 days after irradiation. Patients diagnosed histopathologically as cutaneous malignant melanoma or angiosarcoma without lymph node or distant metastasis are eligible for participation in the trial. The trial protocol is registered with *ClinicalTrials.gov* (NCT04293289) and *JapicCTI* (JapicCTI-195062). Although the clinical trial duration was up to 180 days, the patients were followed up after the end of the clinical trial period. The clinical trial is being conducted in accordance with the Declaration of Helsinki and Good Clinical Practices and compliance with the registered protocol. Institutional review boards at NCCH approved the clinical trial (T4725). Prior to treatment, all patients provided written informed consent. The detail of the clinical trial overview was also found in the previous report (6).

### Case presentation

**Figure 1:**
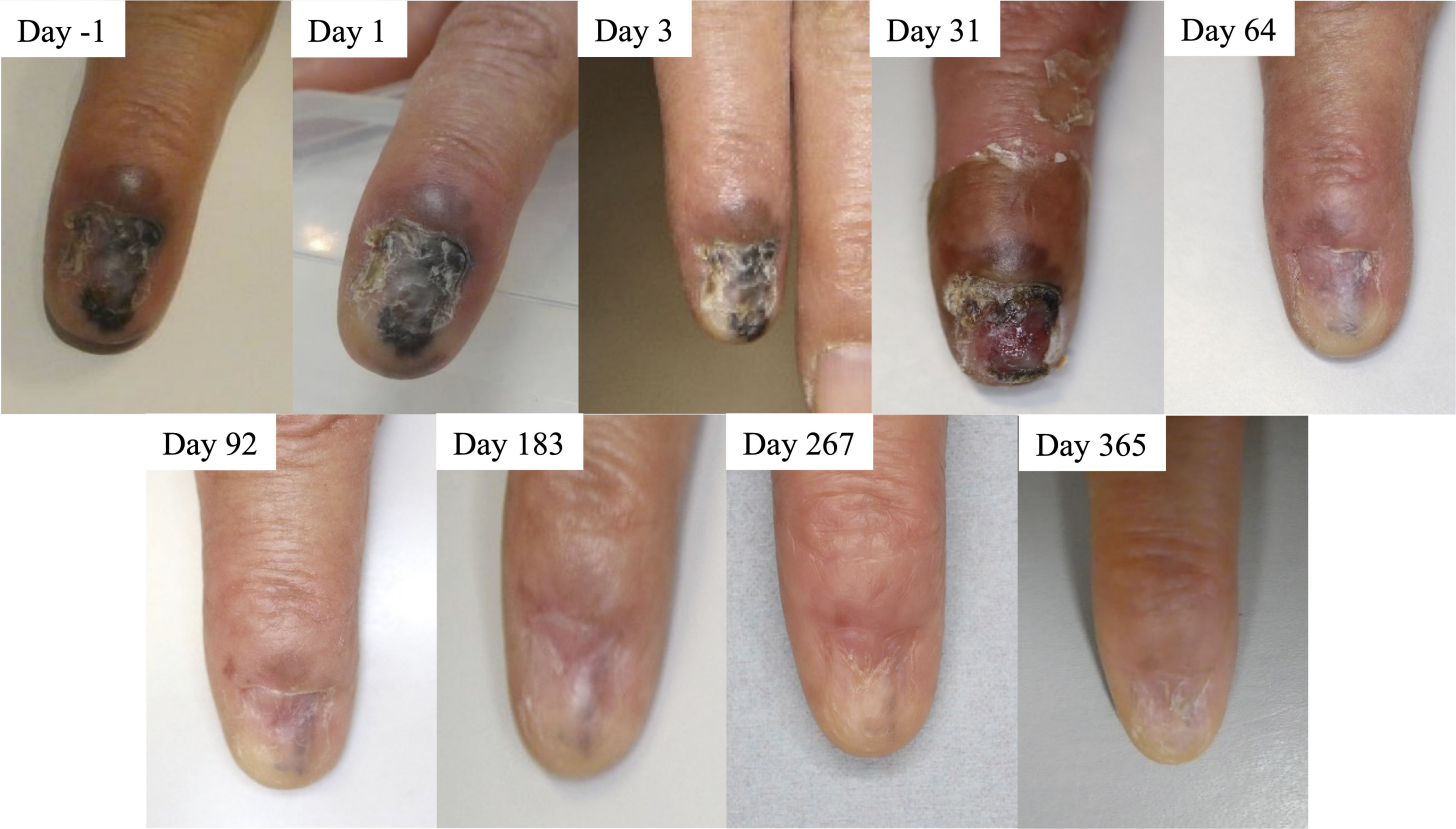
Time course of patient’s tumor before and after boron neutron capture therapy (BNCT). Clinical photos were obtained before treatment start (day -1) and on treatment days 1, 3, 31, 64, 92, 183, 267, and 365. The patient achieved PR day 183 and CR day 365, according to the RECIST 1.1 criteria.

A patient, a previously healthy 72-year-old female, was referred to us for the evaluation of malignant melanoma *in situ on* a second finger of the left hand (**Figure 1**). She was diagnosed with cutaneous malignant melanoma and had no lymph node nor distant metastasis, as determined by standard diagnostic and staging procedures. The tumor size was 20 × 12 mm. Before BNCT, the nail on the left hand’s second finger had been peeled off spontaneously. She declined radical surgery due to its invasiveness and expected aesthetic results, so she was referred for BNCT.

### Treatment

**Figure 2:**
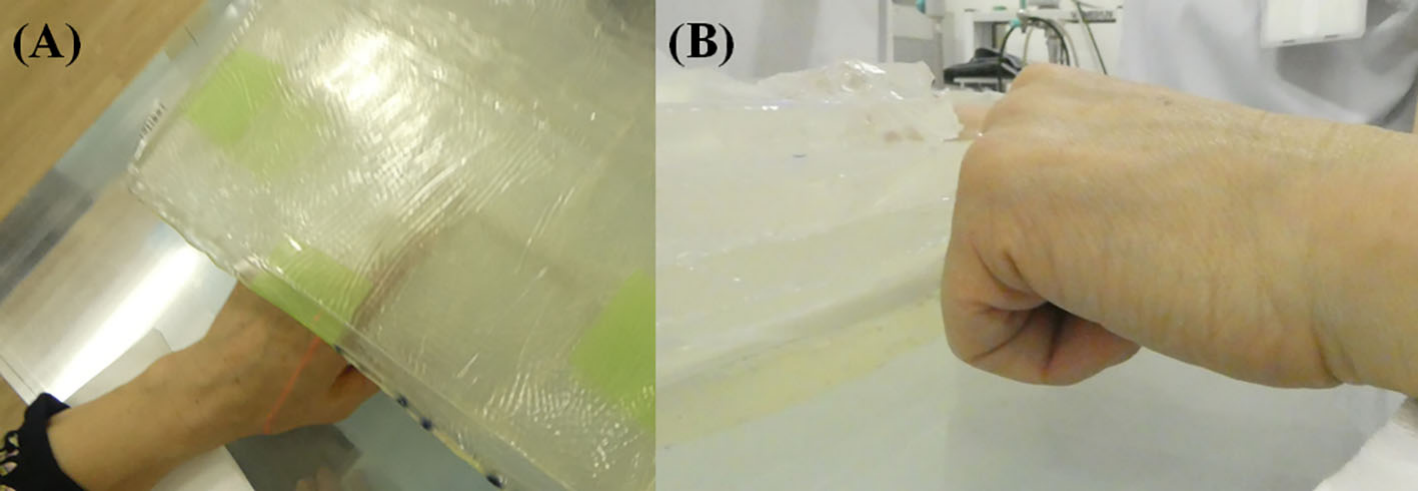
Treatment geometry photos taken at **(A)** corresponding to the neutron beam axis and **(B)** perpendicular to the neutron beam axis. The affected finger was surrounded by the bolus and the other fingers were flexed.

Two-hundred mg/kg/h of SPM-011 was continuously administered intravenously to the patient for 2 hours before neutron irradiation. After this 2-hour administration, the blood was taken to determine the ^10^B concentration in blood using an inductivity coupled plasma optical emission spectrometer (SPS3500DD, Hitachi High-Tech Science Corporation, Tokyo, Japan). After them without a pause, the continuous intravenous administration speed of SPM-011 was decreased to 100 mg/kg/h for the next 1 hour (6). Based on the ^10^B concentration measurement result, the required proton charge was determined to deliver the maximum dose of 18 Gy-Eq to the skin. The neutron irradiation was then performed during the last 1-hour administration. When the neutron irradiation to the patient was complete, the administration of SPM-011 was stopped. At the neutron irradiation, to reduce the delivered dose except for the affected finger, the fingers were flexed except for sticking the affected finger out, and the treatment was then performed. **Figure 2** shows the treatment geometry photo. The affected finger was surrounded by the bolus to achieve the optimal dose.

The total physical BNCT dose, which was comprised of the boron, nitrogen, hydrogen, and gamma-ray dose, was calculated by Monte Carlo Simulation (Particle and Heavy Ion Transport code System (PHITS), ver. 3.020) (12). It was noted that the assumed boron concentration in the blood on treatment planning was 25 ppm. Considering the compound biological effectiveness in the boron dose and the relative biological effectiveness (RBE) in each dose component (**Table 1**), the maximum and minimum expected RBE-weighted doses to the gross tumor volume in the patient were expected as 61.0 and 53.1 Gy-Eq, respectively. The expected RBE-weighted boron, nitrogen, hydrogen, and gamma-ray doses to the skin were then expected as 14.2, 1.2, 0.2, and 2.4 Gy-Eq, respectively. Furthermore, the some difference on the blood boron concentration may be observed between the treatment planning and the actual treatment. In this case, the boron dose is re-calculated to reflect the actual patient boron concentration, and the final dose is re-prescribed to follow the clinical trial protocol. As a result, the actual required proton charges and the actual irradiation time are derived. A detailed description of the dose calculation method was reported in the previous study (6).

**Table 1:** Compound biological effectiveness, relative biological effectiveness, and boron concentration parameters in the dose calculation.

	CBE	RBE (Nitrogen)	RBE (Hydrogen)	RBE (gamma-ray)	Tissue-to-blood ratio of boron concentration
Tumor	4.0 (4)	3.0 (2)	3.0 (13)	1.0	3.5 (4)
Skin	2.5 (4)	3.0 (2)	3.0 (13)	1.0	1.4 (14)

CBE, Compound biological effectiveness; RBE, relative biological effectiveness.

## Results

All data are current as of January 27, 2023.

### Treatment delivered

The treatment was performed on January, 2022. The radiation dose was prescribed so that the maximum dose delivered to the skin was 18 Gy-Eq, which was the last radiation dose level in this trial. The patient received the first patient treatment for cutaneous malignant melanoma using an accelerator-based BNCT system. After the first 2-hour administration of SPM-011, the blood boron concentration measured was 33.7 ppm. Based on the measured boron concentration, the required proton charge and the expected irradiation time were 18230 mC and 30.4 minutes, respectively. The epithermal neutron of 9.7×10^11^ cm^-2^ was provided by CICS-1 (5).

### Clinical outcomes

The epithelial tumor shrank very slowly and reduced in blackness. The patient reached PR on day 183 and CR on day 365 (**Figure 1**). No disease recurrence was noted over the follow-up period of 12 months without any treatment-related grade 2 or higher adverse events. **Table 2** summarizes treatment-related toxicities. Treatment-related grade 1 adverse events of edema on the back of left hand, nausea, radiation dermatitis of left hand, left finger aching pain, dry skin, and skin hyperpigmentation were noted on day 1, 1, 8, 8, 20, and 20, respectively. The skin hyperpigmentation and the other adverse events were relieved within 12 and 3 months, respectively, without any treatment.

**Table 2:** Treatment-related toxicities after boron neutron capture therapy.

	Worst date (Grade)	Recovered date (Grade)
Radiation dermatitis of left hand	Day 8 (G1)	Day 64 (G0)
Dry skin	Day 20 (G1)	Day 92 (G0)
Skin hyperpigmentation	Day 20 (G1)	Day 365 (G0)
Edema on back of left hand	Day 1 (G1)	Day 31 (G0)
Nausea	Day 1 (G1)	Day 31 (G0)
Left finger aching pain	Day 8 (G1)	Day 31 (G0)

## Discussion

This report presents the first patient treatment for cutaneous malignant melanoma treated with an accelerator-based BNCT system. The patient had favorable short-term tumor responses and safe outcomes.

While our follow-up period was relatively short, no progression was observed in the patient. According to the previous report, 28.6% and 47.6% in the recurrent patient group experienced recurrence within 6 and 12 months after surgery although the recurrence pattern of cutaneous malignant melanoma varied depending on the patient’s background (15). Therefore, this case report shows that the accelerator-based BNCT may become a novel treatment option for cutaneous malignant melanoma.

Grade 2 or higher adverse event related to BNCT was not observed in the patient. Those adverse events can also be observed after the conventional radiotherapy, and their severities were similar to milder compared with conventional radiotherapy. While previous studies reported that grade 3 adverse event of a transient asymptomatic hyperamylasemia was frequently observed in patients with head and neck cancer and scalp angiosarcoma after BNCT, it was not observed in this patient (4, 6). It may relate to a tumor location. The previous report investigated hyperamylasemia after the conventional radiotherapy dose to the parotid glands greater than 0.5–1 Gy (16). In this patient, the epithelial tumor was located on the second finger of the left hand. Therefore, radiation exposure to the salivary glands was likely lower than those patients with head and neck cancer and scalp angiosarcoma.

Previous report investigated that the differences between the reactor-based and the accelerator-based BNCT (17). The energy and intensity of neutrons are adjusted to be optimal for BNCT in the accelerator-based BNCT. Thus, previous reports indicated that RBE value of neutron beam in the accelerator-based BNCT was lower than that in the reactor-based BNCT (13, 17, 18). Furthermore, the accelerator-based BNCT also expects shorter irradiation time. However, our clinical trial protocol was developed based on the clinical trials conducted in the reactor-based BNCT, and the delivered dose to a patient was set to be comparable to the clinical study from the reactor-based BNCT (2, 3, 19). As a result, skin reaction from the accelerator-based BNCT was very mild as well as the reactor-based BNCT. Thus, it might be a clinically undetectable difference even if there was a slight different between the reactor-based and the accelerator-based BNCT. Furthermore, previous reports from reactor-based BNCT had manifested the favorable clinical outcome of malignant melanoma (Response rate: 69-95%) (2, 3, 19). Even with this limitation, we expect the accelerator-based BNCT to become a promising option for treating cutaneous malignant melanoma. Furthermore, depending on the progression of the primary lesion, performing sentinel lymph node biopsy and adjuvant therapy may make BNCT a more suitable treatment while this case did not perform. We hope that further cases will reveal optimal pre- and post-treatment strategies for acral cutaneous malignant melanoma treated with the accelerator-based BNCT.

In the conclusion, this case report shows that the accelerator-based BNCT may become a promising treatment modality for cutaneous malignant melanoma. We expects further clinical trials to reveal the efficacy and safety of the accelerator-based BNCT for cutaneous malignant melanoma.

## Data availability statement

The original contributions presented in the study are included in the article/supplementary material. Further inquiries can be directed to the corresponding author.

## Ethics statement

The clinical trial is being conducted in accordance with the Declaration of Helsinki and Good Clinical Practices and compliance with the registered protocol. Institutional review boards at NCCH approved the clinical trial (T4725). Prior to treatment, all patients provided written informed consent. The studies were conducted in accordance with the local legislation and institutional requirements. The participants provided their written informed consent to participate in this study. Written informed consent was obtained from the individual(s) for the publication of any potentially identifiable images or data included in this article.

## Author contributions

HI: Data curation, Funding acquisition, Supervision, Writing – original draft, Writing – review & editing. SN: Formal Analysis, Funding acquisition, Validation, Writing – origBinal draft, Writing – review & editing. NY: Data curation, Resources, Writing – review & editing. ToK: Data curation, Resources, Writing – review & editing. MT: Data curation, Formal Analysis, Resources, Writing – review & editing. TaK: Data curation, Resources, Writing – review & editing. NM: Data curation, Resources, Writing – review & editing. KN: Data curation, Resources, Writing – review & editing. TN: Formal Analysis, Resources, Writing – review & editing. HO: Formal Analysis, Resources, Writing – review & editing. KIi: Formal Analysis, Resources, Writing – review & editing. TC: Formal Analysis, Resources, Writing – review & editing. HirN: Formal Analysis, Resources, Writing – review & editing. AN: Data curation, Resources, Writing – review & editing. MS: Data curation, Resources, Writing – review & editing. KT: Data curation, Resources, Writing – review & editing. KIn: Data curation, Resources, Writing – review & editing. KO: Data curation, Resources, Writing – review & editing. YN: Data ;curation, Resources, Writing – review & editing. KS: Supervision, Writing – review & editing. HitN: Supervision, Writing – review & editing. JI: Supervision, Writing – review & editing, Project administration.
